# Vaccine practices, literacy, and hesitancy among parents in the United Arab Emirates

**DOI:** 10.1371/journal.pone.0307020

**Published:** 2024-08-27

**Authors:** Hiba Jawdat Barqawi, Kamel A. Samara, Samah Mohamed Kannas, Omar Habbal, Nada A. AlSarraf, Mohammad A. Dreezi, Eman Abu-Gharbieh

**Affiliations:** 1 College of Medicine, University of Sharjah, Sharjah, United Arab Emirates; 2 Research Institute of Medical and Health Sciences, University of Sharjah, Sharjah, United Arab Emirates; 3 School of Pharmacy, The University of Jordan, Amman, Jordan; Regional Health Care and Social Agency of Lodi, ITALY

## Abstract

**Background:**

Immunisation is one of public health’s greatest success stories, yet, annually, 20 million children miss out entirely or partially on routine immunisation. National immunisation estimates have the United Arab Emirates (UAE) lagging behind with 4% of children under the age of 1 not having received any vaccines. Vaccine hesitancy is considered one of the biggest barriers to vaccination. This study aims to evaluate the UAE’s parents’ vaccination attitudes and practices as well as estimate vaccine hesitancy’s prevalence and determinants.

**Methodology:**

This cross-sectional, descriptive study collected data from parents across the UAE during the months of March and April 2024. The 60-item questionnaire included the Parental Attitudes towards Childhood Vaccines scale (PACV), the Vaccine Hesitancy Scale, and the Digital Vaccine Literacy (DVL) scale. Univariate, bivariate (chi-squared test), and multivariate (logistic regression) analyses were conducted.

**Results:**

A total of 550 responses were retained. 84.55% of participants were female (n = 465/550), half were middle-aged (31–45 years old), and 21.09% (n = 116/550) were healthcare workers. 94.36% (n = 519/550) had their child/children receive all mandated vaccines. Only 39.82% (n = 219/550) found their level of knowledge about childhood vaccinations to be good/excellent. 70.11% (n = 386/550) of participants had high digital vaccine literacy. More than 95% had positive attitudes towards measles, meningitis, and pertussis vaccines. 14.00% (n = 77/550) were identified as vaccine-hesitant according to the PACV. Overall, using general practitioner/ paediatrician as a knowledge source, digital vaccine literacy, perceived children’s vaccine knowledge, and nationality were associated with lower vaccine hesitancy status.

**Conclusion:**

Vaccine hesitancy exists and is prevalent in the UAE; however, the majority of participants reported high trust in vaccines, the local healthcare systems and physicians. Vaccine hesitancy can be tackled but will require tailored solutions and proactive healthcare workers.

## Introduction

Immunisation, one of public health’s greatest success stories, is responsible for saving millions of lives every year. Yet nearly 20 million infants every year have insufficient access to vaccines with progress being reversed in some countries [1]. In 2012, the World Health Organization (WHO) established the Strategic Advisory Group of Experts on Immunization (SAGE) who began working towards addressing vaccine hesitancy. SAGE defines vaccine hesitancy as “a delay in acceptance or refusal of vaccines despite availability of vaccination services,” proposing the 3C framework for vaccine hesitancy: confidence, complacency, and convenience [2]. The 3C model was updated and expanded in 2018, yielding the 5C model: confidence, complacency, constraints, calculation and collective responsibility [3]. The COVID-19 pandemic itself also disrupted vaccine efforts, as well as exposed weaknesses and exacerbated strains in healthcare systems in 2020 and 2021, leading to setbacks globally [4]. However, continuous efforts have helped the immunisation coverage to begin recovering, with the World Health Assembly endorsing a global strategy (the Immunization Agenda 2030) that envisions a world where everyone at every age can fully benefit from vaccines [5]. In fact, while the number of zero-dose children (children missing out on any vaccination) dropped 3.9 million to reach 14.3 million in 2022, it is still above the pre-pandemic level of 12.9 million [4].

As recognized by the WHO previously, vaccine hesitancy has emerged as one of the most serious threats to global health; unvaccinated individuals lead to failure of herd immunity and can act as virus reservoirs, cause outbreaks, delay spread control [3]. Vaccination attitudes, contrary to previous beliefs, are multifactorial and complex, influenced by a number of individual, contextual, and vaccine-specific factors. For example, studies have shown that individual factors such as personal beliefs, past experiences with vaccines, and perceived risks of vaccination play significant roles in shaping vaccination attitudes [6]. Contextual factors, including socio-economic status, cultural norms, and access to healthcare services, also influence vaccination decisions within communities [7]. Moreover, vaccine-specific factors such as the perceived effectiveness and safety of vaccines, as well as the presence of misinformation or vaccine controversies, further contribute to the complexity of vaccination attitudes [8]. As such, more recent literature has moved to recognizing that vaccine hesitancy cannot be resolved with a “one-size fits all” approach but requires a multimodal and tailored solution targeted to specific populations [9,10]. Moreover, the challenges posed by vaccine hesitancy were further compounded by the emergence of the COVID-19 pandemic. Tackling vaccine hesitancy is possible and ensuring widespread vaccine acceptance is achievable but requires immediate responsiveness towards emerging concerns [11].

According to the United Nations Children’s Fund (UNICEF), in the Middle East and North Africa, 3.8 million children have missed out entirely or partially on routine immunisation between 2019 and 2021. There are significant differences between countries within the MENA region;data from the WHO indicates that some countries in the Gulf Cooperation Council (GCC), such as the UAE and Qatar have relatively high immunization rates compared to other countries in the region, such as Yemen and Syria, where conflict and humanitarian crises have severely disrupted healthcare systems and access to immunization services [4]. Still, the UAE seems to lag behind other countries in the region according to national immunisation estimates, with 4% of children under the age of 1 not having received any vaccines [1]. However, there is a paucity of results; local research in the UAE highlight a mix of positive attitudes and hesitancy towards vaccination among parents, underscoring the need for further investigation [12,13]. Yet, more recent studies have examined factors influencing vaccine acceptance among university students and the general population, shedding light on misinformation, digital literacy, and healthcare professional guidance [14,15]. Given the diverse cultural landscape of the United Arab Emirates (UAE), it is hypothesized that parental attitudes towards childhood vaccination and vaccine hesitancy will vary significantly across demographic factors such as age, educational level, and nationality as well as other variables such as knowledge source and vaccine literacy. As such, this study aims to thoroughly evaluate the UAE’s parents’ general attitudes and vaccination practices, digital vaccine literacy, as well as estimate the prevalence of vaccine hesitancy and its determinants.

## Methodology

### Study population and data collection

This UAE cross-sectional study collected data from parents across the country from 18th March 2024 to 9th April 2024 through convenience sampling. Participants were approached through WhatsApp groups and other social media platforms such as X and Instagram. The UAE is one of the world’s largest consumers of social media and has a nearly 100% internet penetration ratio; as such, the majority of the population can be reached through social media. Only parents with current or previous children were included. A minimum sample size of 385 participants was calculated using Cochran’s sample size formula, assuming a confidence level of 95%, sampling error of 5%, and a standard error of 1.96. A total of 550 responses were retained after removing those not meeting inclusion criteria. The inclusion criteria included English-speaking and/or Arabic-speaking parents living in the UAE, with at least one child. A participant information sheet (PIS) was presented before starting the study and filling the questionnaire indicated consent to participate in the study. Finally, the collected data, which does not contain any identifying information, was stored securely and accessible only by the investigators to ensure confidentiality. The raw unprocessed data can be found in the (S1 File).

### Questionnaire development

The tool used in this study was developed by adapting the questionnaire used by Voo et al. [16] as well as including the following: the Parental Attitudes towards Childhood Vaccines scale (PACV) by Opel et al. [17], the WHO’s Vaccine Hesitancy Scale (VHS) by Larson et al. [18], and the Digital Vaccine Literacy (DVL) scale by Montagni et al. [19]. The questionnaire was originally developed in English and then translated to Arabic. With regards to the PACV, the already translated and validated Arabic version by Alsuwaidi et al. [12] was used. The overall Arabic questionnaire was reviewed multiple times to ensure consistency with the original. Both were pilot tested two times; all provided feedback was evaluated and incorporated if appropriate. Required edits included expanding the options for some of the demographic questions; the VHS, PACV, and DVL were unchanged. The 60-item self-administered questionnaire consisted of three different sections: demographics, childhood vaccination attitudes and knowledge sources, and childhood vaccinations and practices. It included a mixture of yes/no questions, 5-item and 4-item likert scales, as well as single and multi-select questions (S2 File). This research was reviewed and approved by the Research Ethics Committee of the University of Sharjah (Reference Number: REC-24-02-26-01-F). It was conducted in accordance with all relevant guidelines and regulations. No identifying information was collected from the participants.

### Statistical analysis

Only the researchers had access to the raw data. Data was exported from Google Forms to CSV format and processed in python-3 using the Matplotlib-v3.3.4, pandas-v1.2.4, and statsmodels-v0.12.2 packages for analysis and interpretation. Missing values were dealt with through pairwise deletion. Frequency distributions were calculated for each categorical variable and 5-item Likert scales were collapsed to tertiary variables as per the scoring guidelines for the PACV and VHS scales (responses that agreed or strongly agreed with vaccine-hesitant statements were coded as hesitant, responses that disagreed or strongly disagreed with vaccine hesitant statements were coded as non-hesitant, and neutral or “I do not know” responses were coded as unsure). Vaccine hesitancy status was determined using the overall PACV scores. All baseline and demographic characteristics were used to outline determinants of vaccine hesitancy. Chi-squared tests were used for bivariate analyses and logistic regression was used for multivariate modelling. P values less than 0.05 were taken to be significant.

## Results

### Demographics

84.55% of participants were female (n = 465/550). Nearly half were middle-aged (31–45 years old) with only 7.85% being young adults (18–30 years). Almost 95% were married and three-quarters of participants were Other (non-Emirati) Arabs. Only 21.09% (n = 116/550) were healthcare workers and 62.73% (n = 345/550) had 3 children or more in the household. The children’s age distribution varied, with more parents having older children and nearly half having children aged 12–18 years. Majority of parents had medically insured all of their children 75.64%, (n = 416/550), with only 13.45% (n = 74/550) reporting none of their children being insured. Table 1 presents all the participants’ demographic data.

**Table 1:** Demographics and baseline characteristics of participants.

Sex—% (n)		Do your children have health insurance? % (n)	
Female	84.55% (n = 465/550)	No, none of them	13.45% (n = 74/550)
Male	15.45% (n = 85/550)	Yes, all of them	75.64% (n = 416/550)
Age—% (n)		Yes, but only some of them	10.91% (n = 60/550)
Young adult (18–30 years)	7.85% (n = 43/548)	Did you take the COVID-19 vaccine? % (n)	
Middle-aged (31–45 years)	54.01% (n = 296/548)	Not received	5.82% (n = 32/550)
Old-aged (46 years and older)	38.14% (n = 209/548)	1 dose/ 2 doses	42.18% (n = 232/550)
Highest degree obtained—% (n)		3 doses or more	52.0% (n = 286/550)
High school or lower	15.45% (n = 85/550)	Did you take the influenza vaccine last year? % (n)	
Diploma/bachelor’s degree	64.91% (n = 357/550)	Yes	24.73% (n = 136/550)
Postgraduate degree (MSc, PhD., etc.) or higher	19.64% (n = 108/550)	No	75.27% (n = 414/550)
Marital status—% (n)		Has your child/children received the vaccines mandated by the Ministry of Health? % (n)	
Married	94.91% (n = 522/550)	Yes, all of them	94.36% (n = 519/550)
Widowed/ divorced	5.09% (n = 28/550)	Yes, but only some of them	3.27% (n = 18/550)
Nationality—% (n)		No, none of them	1.27% (n = 7/550)
Non-Arab	8.36% (n = 46/550)	Not sure	1.09% (n = 6/550)
Other Arab	75.45% (n = 415/550)	Has your child/children received vaccines other than those mandated by the Ministry of Health? % (n)	
UAE national	16.18% (n = 89/550)	Yes, all of them	31.99% (n = 175/547)
Place of Residence—% (n)		Yes, but only some of them	12.43% (n = 68/547)
Abu Dhabi	60.91% (n = 335/550)	No, none of them	44.61% (n = 244/547)
Dubai	13.27% (n = 73/550)	Not sure	10.97% (n = 60/547)
Sharjah and other northern emirates	25.82% (n = 142/550)	How regularly do you visit your children’s doctor for checkups? % (n)	
Field of work—% (n)		I do not regularly visit	35.27% (n = 194/550)
Non-healthcare worker	33.27% (n = 183/550)	Every month	4.36% (n = 24/550)
Healthcare (doctor, nurse, dentist, pharmacist, etc.)	21.09% (n = 116/550)	Every 3 months	10.0% (n = 55/550)
Housewife	40.55% (n = 223/550)	Every 6 months	12.91% (n = 71/550)
Unemployed	5.09% (n = 28/550)	Annually	24.18% (n = 133/550)
Number of children in household—% (n)		Other	13.27% (n = 73/550)
1	13.64% (n = 75/550)	I find my level of knowledge about childhood vaccinations to be—% (n)	
2	23.64% (n = 130/550)		
3	23.09% (n = 127/550)	Poor/ inadequate	21.45% (n = 118/550)
4 or more	39.64% (n = 218/550)	Adequate	38.73% (n = 213/550)
Age of children in household—% (n)		Good/ excellent	39.82% (n = 219/550)
1 months—11 months	7.64% (n = 42/550)	Do you think you have enough sources of information on immunization? % (n)	
1 years—2 years	10.36% (n = 57/550)		
3 years—5 years	26.55% (n = 146/550)	Yes	64.0% (n = 352/550)
6 years—11 years	40.18% (n = 221/550)		
12 years—18 years	52.36% (n = 288/550)	No	36.0% (n = 198/550)
19 years or older	33.45% (n = 184/550)		

### Vaccination practices and knowledge

75.27% (n = 414/550) of participants reported receiving the influenza vaccine last year, compared to nearly 95% that reported receiving the COVID-19 vaccine (41.18% (n = 232/550) receiving 1 or 2 doses and 52.00% (n = 286/550) receiving 3 doses or more). More than a third of parents did not regularly visit their children’s doctor for checkup. While 94.36% (n = 519/550) had their child/children receive all vaccines mandated by the Ministry of Health, only 31.99% (n = 175/547) had their child/children receive vaccines other than those mandated. Table 1 contains additional information regarding the parent’s practices.

**Fig 1:**
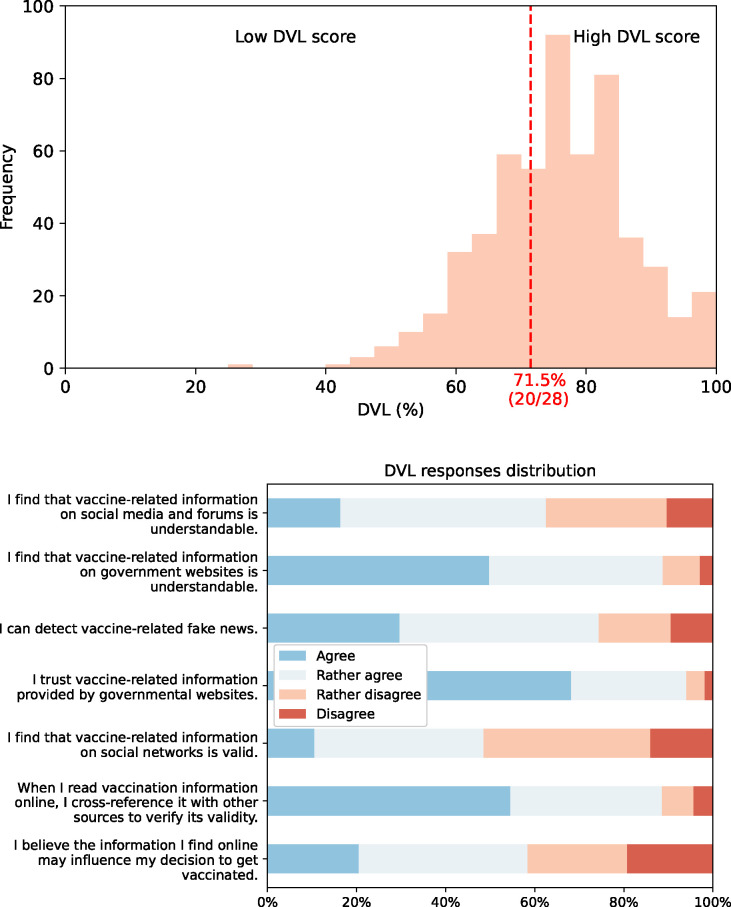
Participant’s Digital Vaccine Literacy (DVL) scores. (a) shows the distribution of the overall DVL scores with the cut-off for low/high digital vaccine literacy. (b) shows the response distribution for each scale item.

Only 39.82% (n = 219/550) found their level of knowledge about childhood vaccinations to be good/excellent, with a fifth reporting poor/inadequate knowledge. Additionally, only 64.00% (n = 352/550) believed they had enough sources of information on immunisation. The most commonly utilised source for information on childhood vaccination was the general practitioner/ primary care paediatrician at 55.64% (n = 306/550). It was followed by governmental websites 36.55%, (n = 201/550), specialist doctors 31.18%, (n = 177/550), and social media 24.73%, (n = 136/550). Table 2 lists the various knowledge sources and the number of participants who utilised them. Digital Vaccine Literacy (DVL) scores were high (*μ* = 75.01, *σ* = 10.85) with the overwhelming majority 70.11%, (n = 386/550) of participants showing high digital vaccine literacy (score ≥ 20/28). Fig 1 displays the distribution of the DVL scores as well as the results of each individual response; most evident is the strong trust in vaccine information provided by governmental websites 94.00%, (n = 517/550). Notably however, the DVL scale showed poor internal consistency (Cronbach’s *α* = 0.53; 95% CI: 0.47–0.59).

**Table 2:** Parent’s vaccination attitudes.

What sources do you use to get information about childhood vaccinations? % (n)					Which of the following are concerns you had/have regarding childhood vaccines? % (n)			
General practitioner/primary care pediatrician		55.64% (n = 306/550)		I have no concerns regarding childhood vaccines.			48.36% (n = 266/550)	
Specialist doctors		32.18% (n = 177/550)		Painful and causes fever.			31.64% (n = 174/550)	
Family health unit		19.82% (n = 109/550)		The ingredients in vaccines are unsafe.			11.27% (n = 62/550)	
Governmental websites (CDC, MOHAP, DHA, WHO, etc.)		36.55% (n = 201/550)		Too many vaccines are given in one doctor’s visit.			4.18% (n = 23/550)	
Blog/ forum/ non-governmental websites		14.36% (n = 79/550)		Vaccines may cause chronic diseases (such as diabetes or asthma).			6.18% (n = 34/550)	
School/university		18.91% (n = 104/550)						
Mass media (radio/tv/newspapers)		20.18% (n = 111/550)		Vaccines are given to prevent diseases that children are not likely to get.			6.0% (n = 33/550)	
Social media		24.73% (n = 136/550)						
Word of mouth		15.82% (n = 87/550)		Vaccines are given to prevent diseases that are not serious.			2.36% (n = 13/550)	
No-vax movements/groups		1.27% (n = 7/550)						
Other		5.45% (n = 30/550)		Vaccines may cause learning disabilities (such as autism).			10.0% (n = 55/550)	
I do not any use any sources		4.0% (n = 22/550)		I have other concerns regarding childhood vaccines.			12.73% (n = 70/550)	
Reasons for delaying a vaccine (PACV—#1) % (n)					Reasons for refusing a vaccine (PACV—#2) % (n)			
I did not delay any vaccines for reasons other than allergy		71.82% (n = 395/550)		I did not refuse any vaccines for reasons other than allergy			77.09% (n = 424/550)	
Concerned about side effects		5.27% (n = 29/550)		Concerned about side effects			6.18% (n = 34/550)	
Forgetfulness		8.55% (n = 47/550)		Forgetfulness			4.91% (n = 27/550)	
Fear of vaccine administration		2.91% (n = 16/550)		Fear of vaccine administration			5.09% (n = 28/550)	
Lack of recommendation		2.91% (n = 16/550)		Lack of recommendation			3.27% (n = 18/550)	
Vaccine was not available		1.82% (n = 10/550)		Vaccine was not available			1.45% (n = 8/550)	
Other		12.36% (n = 68/550)		Other			8.18% (n = 45/550)	
How important, if at all, do you think it is for children to be vaccinated against each of the following—% (n)								
Disease	Do not know	Not at all important	Not very important			Fairly important		Very important
Measles	3.27% (n = 18/550)	0.18% (n = 1/550)	1.27% (n = 7/550)			10.91% (n = 60/550)		84.36% (n = 464/550)
Meningitis	3.09% (n = 17/550)	0.18% (n = 1/550)	1.45% (n = 8/550)			7.64% (n = 42/550)		87.64% (n = 482/550)
Pertussis	4.73% (n = 26/550)	0.18% (n = 1/550)	2.0% (n = 11/550)			12.91% (n = 71/550)		80.18% (n = 441/550)
Rotavirus	15.09% (n = 83/550)	0.91% (n = 5/550)	3.82% (n = 21/550)			15.45% (n = 85/550)		64.73% (n = 356/550)
COVID-19	7.27% (n = 40/550)	13.27% (n = 73/550)	18.55% (n = 102/550)			24.0% (n = 132/550)		36.91% (n = 203/550)
Influenza	3.82% (n = 21/550)	10.36% (n = 57/550)	25.45% (n = 140/550)			32.18% (n = 177/550)		28.18% (n = 155/550)

### General vaccination attitudes

Table 2 presents the results regarding some general participants’ vaccination attitudes. 48.36% (n = 266/550) of parents had no concerns regarding childhood vaccines; with 31.64% (n = 174/550) concerned about possible pain and fever post-vaccination. No predominant concern dominated: 11.27% worried that ingredients in vaccines were unsafe (n = 62/550), 10.00% (n = 55/550) believed that vaccines may cause learning disabilities such as autism, the remaining concerns were infrequent. Overall, 71.82% (n = 395/550) did not delay any vaccines (for reasons other than allergy) and 77.09% (n = 424/550) did not refuse any vaccines (for reasons other than allergy). As with concerns, no predominant reason emerged for delaying or refusing vaccines. Concerns about side-effects were reported by 5.29% (n = 29/550) of those delaying vaccines and 6.18% (n = 34/550) of those refusing vaccines. Participants were also asked about the importance of vaccination against a number of well-known diseases. Results were overwhelmingly positive for measles, meningitis, and pertussis with more than 95% regarding vaccination against those illnesses as fairly/ very important. Rotavirus perceived vaccine importance dropped with 80.18% (n = 441/550) finding it to be fairly/very important. Finally, COVID-19 and influenza vaccines were viewed as less important by the participants: 31.82% (n = 175/550) and 35.81% (n = 197/550) found the vaccines to be not at all/not very important, respectively.

### Vaccine hesitancy

**Fig 2:**
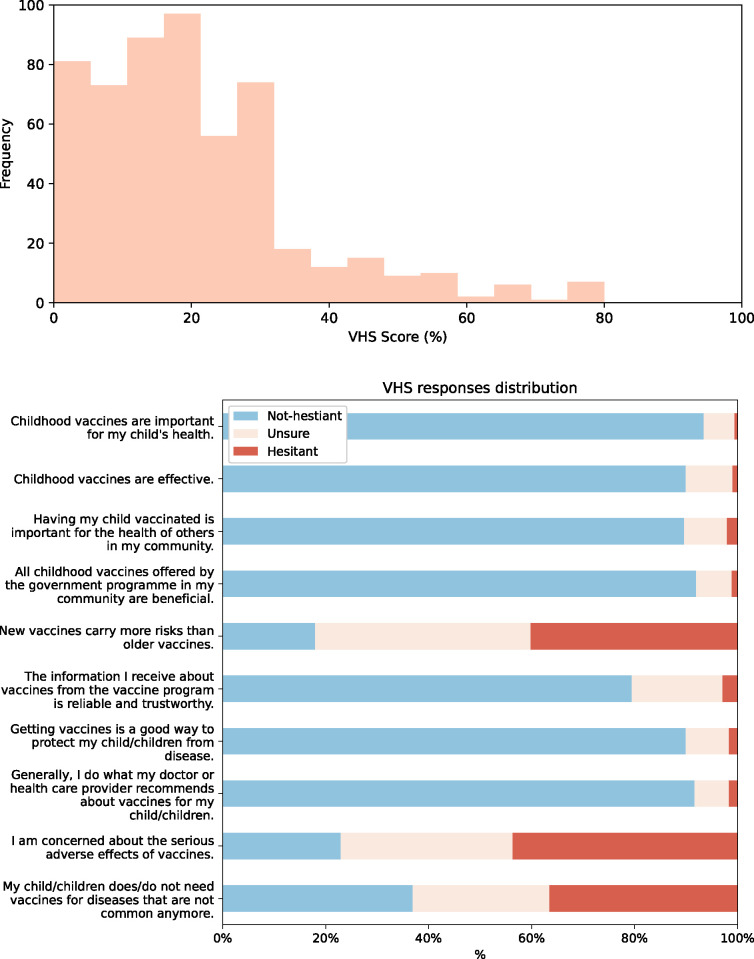
Participant’s Vaccine Hesitancy Scale (VHS) scores. (a) shows the distribution of the overall VHS scores while (b) shows the response distribution for each scale item.

**Fig 3:**
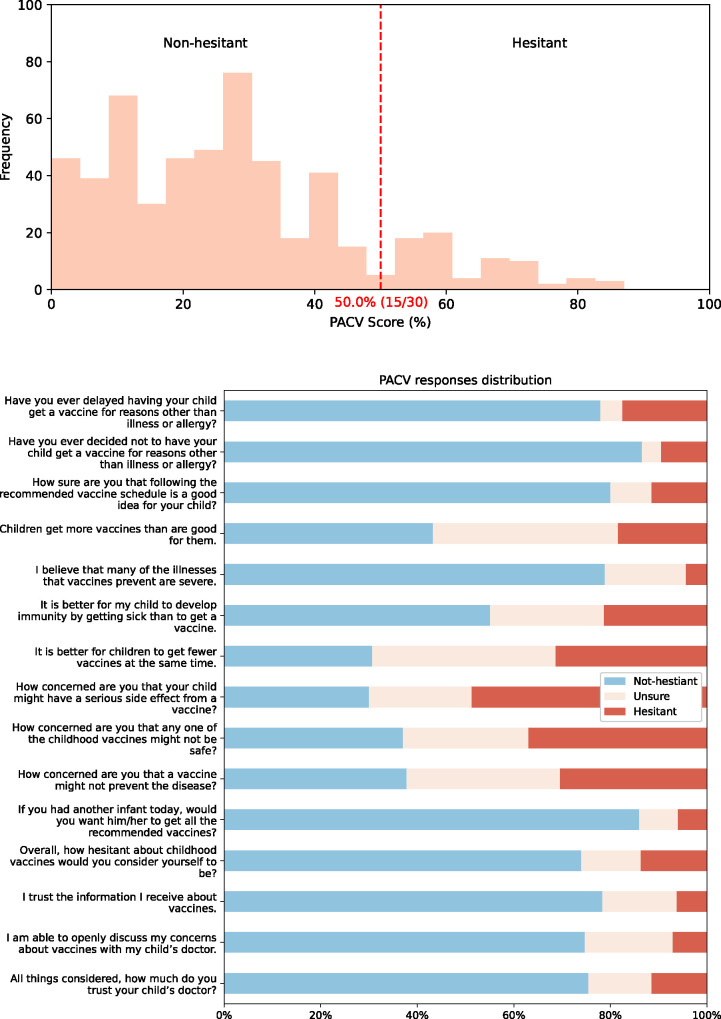
Participant’s Parental Attitudes Towards Childhood Vaccines (PACV) scores. (a) shows the distribution of the overall PACV scores with the cut-off for non-hesitant/hesitant. (b) shows the response distribution for each scale item.

Two scales were used to evaluate vaccine hesitancy: WHO’s Vaccine Hesitancy Scale (VHS) and the Parental Attitudes towards Childhood Vaccines (PACV), shown in Figs 2 and 3. respectively. VHS scores showed low hesitancy overall (*μ* = 21.35%, *σ* = 14.92%). The scale measures two underlying factors: lack of trust and perceived vaccine risk. Fig 2. highlights how participants overwhelmingly scored high on trust (less than 3% displayed hesitant attitudes across items #1–4,6–8) but displayed hesitancy when it came to perceived risk (around two-fifths had hesitant attitudes for items #7,9,10). Unlike the PACV, there is no well-established cut-off for VHS scores to categorize parents into vaccine hesitant and non-hesitant. PACV scores tended to be higher than VHS scores (*μ* = 27.59%, *σ* = 18.52%), with only 14.00% (n = 77/550) meeting the traditional 50% cut-off for being hesitant (with only 67.53% (n = 52/77) recognizing themselves as vaccine-hesitant). The highest level of vaccine trust was seen in PACV item #2 with 86.55% showing non-hesitant attitudes towards refusing vaccines. Additionally, parents had strong positive attitudes towards their child/children’s doctor with nearly three-quarters finding them to be trustworthy and reporting being able to discuss all their concerns with the doctors.

PACV was used to determine the overall vaccine hesitancy status of the parents. Only using general practitioner/ paediatrician as a knowledge source, digital vaccine literacy, perceived children’s vaccine knowledge, and nationality were found to be significant at the bivariate level (using chi-squared). The four determinants were fed into a multivariate logistic regression model, the results of which can be seen in Table 3. Overall, having a general practitioner/ paediatrician knowledge source (OR: 0.288, 95% CI: 0.167–0.495), adequate (OR: 0.536, 95% CI: 0.289–0.995) or good/excellent (OR: 0.374, 95% CI: 0.193–0.724) perceived children’s vaccine knowledge, other (non-Emirati) Arab (OR:0.318, 95% CI: 0.145–0.695), and having high digital vaccine literacy (OR:0.436, 95% CI: 0.257–0.739) were associated with lower vaccine hesitancy status.

**Table 3:** The results of the logistic regression modeling the determinants of being vaccine hesitant. P values for the bivariate chi-squared tests are reported below each variable. Rows with significant p values are bolded. OR: Odds ratio; CI: Confidence interval; SE: Standard error.

**Predictors of being vaccine hesitant–Binary Logistic Regression (LR)**							
**Model Terms**		$OR\;(e^{\beta_{i}})$	**95% CI for OR**	**SE**	**z-Statistic**		**P value**
**Intercept (*β*_0_)**		**1.940**	**0.791–4.759**	**0.458**	**1.447**		**0.148**
**General practitioner/ pediatrician knowledge source** **(P value: < 0.0005)**	Does not use	-	-	-	-	-	
	**Uses**	**0.288**	**0.167–0.495**	**0.276**	**-4.507**	**<0.0005**	
**Perceived children’s vaccine knowledge** **(P value: < 0.0005)**	Poor/ inadequate	-	-	-	-	-	
	**Adequate**	**0.536**	**0.289–0.995**	**0.316**	**-1.974**	**0.048**	
	**Good/ excellent**	**0.374**	**0.193–0.724**	**0.338**	**-2.915**	**0.004**	
**Nationality** **(P value: < 0.0005)**	Non-Arab	-	-	-	-	-	
	**Other Arab**	**0.318**	**0.145–0.695**	**0.399**	**-2.874**	**0.004**	
	UAE national	0.900	0.370–2.186	0.453	-0.233	0.816	
**Digital Vaccine Literacy** **(P value: 0.002)**	Low	-	-	-	-	-	
	**High**	**0.436**	**0.257–0.739**	**0.270**	**-3.079**	**0.002**	
**Log-Likelihood: -194.65**	**Log-Likelihood of Null Model: -222.73**		**Log-Likelihood Ratio P value: <0.0005**				

## Discussion

### Demographic influences on vaccine hesitancy

This study found 14% of the participants to be vaccine hesitant, the majority of which self-reported as such. Moreover, even though the majority reported high trust in the local healthcare systems and physicians, parents still tended to have non-negligible concerns and worry regarding vaccine side-effects, ingredients, and effectiveness. Classical and well-established vaccines were viewed favourably by nearly everyone while newer vaccines were subject to more scepticism. The majority of participants were also found to have high digital vaccine literacy, even though less than half recognized their knowledge of childhood vaccines to be good/excellent. Finally, the study also recognized four demographic factors that were associated with lower rates of vaccine hesitancy, specifically using general practitioner/ paediatrician as a knowledge source, high digital vaccine literacy, high perceived children’s vaccine knowledge, and being non-Emirati Arab were associated with lower vaccine hesitancy. Surprisingly, a number of demographics that were hypothesized to influence vaccine hesitancy were found to be unassociated with the measure; yet, as expected more knowledgeable parents with higher digital vaccine literacy did have more positive parental attitudes toward children vaccination.

Vaccines are essential. Between 2010 and 2018, the measles vaccine alone prevented 23 million deaths annually. However, progress in some countries has stalled or even reversed, undermining past achievements and increasing the risk of outbreaks [5]. Given how large of a threat vaccine hesitancy has become, evaluating and monitoring hesitancy is essential as spikes in vaccine hesitancy have been much more extreme, in part due to the decline in public’s trust of experts, rise of political polarisation, belief-based extremism, and preference for alternative health [11]. The Gulf Cooperation Council (GCC) countries, including the UAE, are no exception and it will be vitally important to understand the population’s concern to be able to tackle vaccine hesitancy, as no one single approach can address and eliminate hesitancy [20]. The results of this study show no clear factor explaining vaccine delaying or refusal, again highlighting the multifaceted nature of vaccine hesitancy and the need for tailored and targeted approaches.

This study addresses a gap highlighted by a recent systematic review which noted that while very little research has been conducted on vaccine hesitancy in the region. The review found that most published work focused on Saudi Arabia and that vaccine hesitancy was prevalent in the GCC, both among the public and healthcare workers [20]. Rabei et al. had initially reported widespread positive attitudes towards vaccines in the UAE coupled with poor knowledge, culminating with a hesitancy rate of 10% [13]. More recently, Alsuwaidi et al. found 12% (n = 36/300) of participants to be vaccine hesitant (according to the PACV). Interestingly, at the bivariate level, they found being male and divorced were associated with increased vaccine hesitancy. Similar to this study, age, educational level, and nationality also had no significant association on hesitancy status [12]. Additionally, and similar to a Saudi study, nearly 95% of participants agreed/strongly agreed that vaccines are important [21]. Yet, parents in general tend to be most concerned regarding negative consequences of vaccines, specifically about vaccines causing illnesses, children receiving too many vaccines, and/or vaccine ingredients being harmful [10].

### Trust in healthcare

This study also showed a high level of trust in the local healthcare system and national immunisation program. This is not surprising given that a high level of trust in the UAE’s healthcare system and physicians has been consistently shown [22,23]. Healthcare providers have been and continue to be the most trusted source about vaccines for the public [10]. Knowledge is an important protective factor against vaccine hesitancy. Voo et al. showed that parents with good vaccine knowledge and awareness were less hesitant, and their children were more likely to have their immunizations be up to date [16]. In fact, a technical report by the European Centre for Disease Prevention and Control (ECDC) found that 27 out of 40 interventions to tackle vaccine hesitancy used dialogue and communication to enhance vaccine trust and health literacy among parents [9]. However, and moving forward, healthcare workers need to adopt a more proactive role in discussing vaccines and their importance, effectiveness, and safety with patients [24].

### The role of social media

The role of social media and vaccine hesitancy is a novel area of research; a scoping review by the ECDC highlighted several ethical issues underpinning this issue as well as lack of standardised method for monitoring and analysing current social media vaccine information [25]. The Centre for Countering Digital Hate (CCDH) reports that anti-vaccination campaigners’ social media accounts have over 62 million followers across the various platforms with annual revenues of $36 million and are worth over $1.1 billion in ad revenue to Big Tech [26]. Additionally, the role of social media in fueling vaccine hesitancy grew substantially during the COVID-19 pandemic. Online social media is rampant with misinformation regarding vaccines, with the situation expected only to get worse with the rise of artificial intelligence systems that can rapidly disseminate false information online that social media bots can amplify [24]. In a systematic review that aimed to identify health misinformation on various social media platforms, vaccines were the most common topic, accounting for nearly two-fifths of all misinformation [27].

## Limitations

Finally, it is important to discuss some of the limitations of this study. The results of this study depended on what the parents reported without independent verification. Moreover, convenience and snowball sampling were used (leading to a female-heavy sample). Additionally, several biases such as social desirability, recall and response bias can affect results. Additional variables can also be explored along with their relationship with vaccine hesitancy such as religious views. However, the survey was distributed among all Emirates and was completely anonymous to ensure authentic and genuine responses. Moreover, the results are in line with what has been reported both regionally and globally.

## Conclusion

Immunisation, one of public health’s greatest success stories, is being threatened by the rise of vaccine hesitancy, both globally and regionally. In the UAE, 14% of the participants were found to be vaccine hesitant. This highlights that even given the massive investments that the UAE government has already poured into tackling hesitancy, a non-negligible proportion remains. However, the majority of participants reported high trust in vaccines as well as the local healthcare systems and physicians, all while having non-negligible concerns and worries regarding vaccine side-effects, ingredients, and effectiveness. This necessitates adopting a public health campaign that continually attempts to address and minimize vaccine hesitancy in the country as well as build on the solid trust in the local health systems. Social media has been a massive force in amplifying and promoting vaccine misinformation. Vaccine hesitancy can be tackled but will require tailored and innovated solutions specific to the UAE population and healthcare workers need to adopt a more proactive role in discussing vaccines and their importance.

## Supporting information

S1 File Raw data. (PDF)

S2 File English and Arabic questionnaires. (XLSX)
