# Supplementary material for: Vaccine practices, literacy, and hesitancy among parents in the United Arab Emirates
Source: PLoS One. 2024 Aug 27;19(8):e0307020. doi: 10.1371/journal.pone.0307020 (PMC11349212; doi:10.1371/journal.pone.0307020)
Supplement: S1 File — (PDF) [file pone.0307020.s001.pdf]

Parental attitudes toward pediatric vaccinations in the UAE.

We are a group of medical students at the University of Sharjah conducting a research project about the “Parental attitudes toward pediatric vaccinations in the UAE." The purpose of this study is to evaluate the attitudes of parents toward childhood vaccinations in the UAE.

You have been randomly selected to participate in this study and your participation is strictly voluntary. If you agree to participate, you will be asked to fill out a questionnaire that will take 5 to 7 minutes of your time.

There are no risks associated with participation in this study. The questionnaire is anonymous, and we assure you that your responses will be confidential and will be used only for research purposes.

You are free to withdraw before and during answering the questionnaire; however, as soon as you submit your response, we will not be able to remove it from our data due to the study’s anonymous nature.

If you have any questions regarding this study, please feel free to contact our Research Supervisor - Dr. Hiba Barqawi at hbarqawi@sharjah.ac.ae or 06-5057268.

In case of concerns or complaints regarding your participation in this research, you can contact the Research Ethics Committee at: 06-5057304 or by Email: REC@sharjah.ac.ae

Thanks in advance for your participation.

لإجابة الإستبيان باللغة العربية اضغط هنا:
https://forms.gle/J716fn7PbH2B1ZNBA

Please check the box below to proceed to the survey:

- I have read the above information and agree to participate in this research.

1) Do you live in the UAE?
   - Yes
   - No
2) Do you have any children (currently and/or from before)?
   - Yes
   - No

Demographics

Please select the option that best fits you.

3) Sex:
   - Male
   - Female
4) Age:
5) Highest Degree Obtained:
   - Middle School or lower
   - High School
   - Diploma/Bachelor's Degree
   - Postgraduate Degree (MSc, PhD., etc.) or higher
6) Marital Status:
   - Married
   - Divorced
   - Widowed
7) Nationality:
   - UAE National
   - Other Arab
   - Non-Arab
8) Place of Residence:
   - Abu Dhabi
   - Dubai
   - Sharjah
   - Ajman
   - Umm Al Quwain

- Ras Al Khaimah
- Fujairah

9) Field of work:
- Healthcare (Doctor, Nurse, Dentist, Pharmacist, Healthcare Administration, etc.) worker
- Non-Healthcare worker
- Student (Health Sciences, Medicine, Dentistry, etc.)
- Student (non-health related majors)
- Housewife
- Unemployed (healthcare background)
- Unemployed (non-healthcare background)

10) Did you take the COVID-19 vaccine?
- No
- Yes, 1 dose
- Yes, 2 doses
- Yes, 3 doses
- Yes, 4 doses or more

11) Did you take the influenza vaccine last year?
- Yes
- No

12) Number of children in household:
- 1
- 2
- 3
- 4 or more

13) Age of children in household:
- 1 months - 11 months
- 1 years - 2 years
- 3 years - 5 years
- 6 years - 11 years
- 12 years - 18 years
- 19 years or older

14) Do your children have health insurance
- Yes, all of them do have insurance
- Yes, but only some of them have insurance

- No, none of them have insurance

15) Has your child/children received the routine childhood vaccinations mandated by the UAE's Ministry of Health?

16) Mandated vaccines are those that are part of the UAE National Immunization Program

- Yes, all of them have received the vaccinations
- Yes, but only some of them have received the vaccinations
- No, none of them have received the vaccinations
- Not sure

17) Has your child/children received vaccines other than those mandated by the UAE's Ministry of Health?

Such vaccines are not part of the UAE National Immunization Program but may be recommended by a physician.

- Yes, all of them have received the vaccinations
- Yes, but only some of them have received the vaccinations
- No, none of them have received the vaccinations
- Not sure

18) Which of the following are concerns you had/have regarding childhood vaccines?

- Painful and causes fever.
- Too many vaccines are given in one doctor's visit.
- Vaccines may cause learning disabilities (such as autism).
- The ingredients in vaccines are unsafe.
- Vaccines are given to children to prevent diseases that are not serious.
- Vaccines are given to children to prevent diseases that they are not likely to get.
- Vaccines may cause chronic diseases (such as diabetes, asthma).
- I have other concerns regarding childhood vaccines.
- I have no concerns regarding childhood vaccines.

19) How regularly do you visit your children's doctor for checkups?

- Every month
- Every 3 months
- Every 6 months

- Annually
- Other
- I don't visit my children's doctor for regular checkups

Childhood vaccination attitudes and knowledge sources

20) How important, if at all, do you think it is for children to be vaccinated against each of the following diseases?

1. Measles:
   - Very important
   - Fairly important
   - Not very important
   - Not at all important
   - Don't know
2. Meningitis:
   - Very important
   - Fairly important
   - Not very important
   - Not at all important
   - Don't know
3. Pertussis:
   - Very important
   - Fairly important
   - Not very important
   - Not at all important
   - Don't know
4. Rotavirus disease:
   - Very important
   - Fairly important
   - Not very important
   - Not at all important
   - Don't know
5. COVID-19:
   - Very important
   - Fairly important
   - Not very important

- Not at all important
- Don't know

6. Influenza:
    - Very important
    - Fairly important
    - Not very important
    - Not at all important
    - Don't know

State the extent to which you **AGREE** or **DISAGREE** with the following statements:

21) Childhood vaccines are important for my child's health.
    - Strongly agree
    - Agree
    - Neutral
    - Disagree
    - Strongly disagree
22) Childhood vaccines are effective.
    - Strongly agree
    - Agree
    - Neutral
    - Disagree
    - Strongly disagree
23) Having my child vaccinated is important for the health of others in my community.
    - Strongly agree
    - Agree
    - Neutral
    - Disagree
    - Strongly disagree
24) All childhood vaccines offered by the government programme in my community are beneficial.
    - Strongly agree
    - Agree
    - Neutral

- Disagree
- Strongly disagree

25) New vaccines carry more risks than older vaccines.
    - Strongly agree
    - Agree
    - Neutral
    - Disagree
    - Strongly disagree
26) The information I receive about vaccines from the vaccine program is reliable and trustworthy.
    - Strongly agree
    - Agree
    - Neutral
    - Disagree
    - Strongly disagree
27) Getting vaccines is a good way to protect my child/children from disease.
    - Strongly agree
    - Agree
    - Neutral
    - Disagree
    - Strongly disagree
28) Generally, I do what my doctor or health care provider recommends about vaccines for my child/children.
    - Strongly agree
    - Agree
    - Neutral
    - Disagree
    - Strongly disagree
29) I am concerned about the serious adverse effects of vaccines.
    - Strongly agree
    - Agree
    - Neutral
    - Disagree
    - Strongly disagree

30) My child/children does/do not need vaccines for diseases that are not common anymore.

- Strongly agree
- Agree
- Neutral
- Disagree
- Strongly disagree

The following questions relate to your knowledge regarding childhood vaccines

31) I find my level of knowledge about childhood vaccinations to be:

- Poor
- Inadequate
- Adequate
- Good
- Excellent

32) Do you think you have enough sources of information on immunization?

- Yes
- No

33) What are sources you use to get information about childhood vaccinations?

- Word of mouth
- General practitioner/primary care pediatrician
- Specialist doctors
- Family Health Unit
- Governmental websites (CDC, MOHAP, DHA, DOH, WHO, etc.)
- Blog/forum/non-governmental websites
- Mass media (radio/TV/newspapers)
- Social media
- School/university
- No-Vax movements/groups
- I do not any use any sources for getting information about childhood vaccinations.
- Other

State the extent to which you **AGREE** or **DISAGREE** with the following statements:

34) I find that vaccine-related information on social media and forums is understandable.
    - o Disagree
    - o Rather disagree
    - o Rather agree
    - o Agree
35) I find that vaccine-related information on government websites is understandable.
    - o Disagree
    - o Rather disagree
    - o Rather agree
    - o Agree
36) I can detect vaccine-related fake news.
    - o Disagree
    - o Rather disagree
    - o Rather agree
    - o Agree
37) I trust vaccine-related information provided by governmental websites.
    - o Disagree
    - o Rather disagree
    - o Rather agree
    - o Agree
38) I trust vaccine-related information provided by doctors.
    - o Disagree
    - o Rather disagree
    - o Rather agree
    - o Agree
39) I find that vaccine-related information on social networks is valid.
    - o Disagree
    - o Rather disagree
    - o Rather agree
    - o Agree

40) When I read vaccination information online, I cross-reference it with other sources to verify its validity.
   - ○ Disagree
   - ○ Rather disagree
   - ○ Rather agree
   - ○ Agree

41) I believe the information I find online may influence my decision to get vaccinated.
   - ○ Disagree
   - ○ Rather disagree
   - ○ Rather agree
   - ○ Agree

Childhood vaccines practices and beliefs

The following are some more questions regarding your beliefs and practices towards childhood vaccines.

For each question, please choose the option that best describes your views, beliefs or practices

42) Have you ever delayed having your child get a vaccine for reasons other than illness or allergy?
   - ○ Yes
   - ○ No
   - ○ I don't know

43) What is the reason for the delay?
   - ○ I did not delay any vaccines for reasons other than allergy or infection
   - ○ Lack of recommendation by the doctor
   - ○ Fear of vaccine administration for your child
   - ○ Forgetfulness
   - ○ Concerned about the side effects
   - ○ Vaccine was not available in the vaccination center
   - ○ Other

44) Have you ever decided not to have your child get a vaccine for reasons other than illness or allergy?
   - ○ Yes

- No
- I don't know

45) What is the reason for not having the child get the vaccine?
- I did not refuse any vaccines for reasons other than allergy or infection
- Lack of recommendation by the doctor
- Fear of vaccine administration for your child
- Forgetfulness
- Concerned about the side effects
- Vaccine was not available in the vaccination center
- Other

46) How sure are you that following the recommended vaccine schedule is a good idea for your child?
- 0 Not sure at all
- 1
- 2
- 3
- 4
- 5
- 6
- 7
- 8
- 9
- 10 Completely sure

47) Children get more vaccines than are good for them.
- Strongly disagree
- Disagree
- Neutral
- Agree
- Strongly agree

48) I believe that many of the illnesses that vaccines prevent are severe.
- Strongly disagree
- Disagree
- Neutral
- Agree

- Strongly agree

49) It is better for my child to develop immunity by getting sick than to get a vaccine.
    - Strongly disagree
    - Disagree
    - Neutral
    - Agree
    - Strongly agree

50) It is better for children to get fewer vaccines at the same time.
    - Strongly disagree
    - Disagree
    - Neutral
    - Agree
    - Strongly agree

51) How concerned are you that your child might have a serious side effect from a vaccine?
    - Very concerned
    - Somewhat concerned
    - Neutral
    - Not too concerned
    - Not concerned at all

52) How concerned are you that any one of the childhood vaccines might not be safe?
    - Very concerned
    - Somewhat concerned
    - Neutral
    - Not too concerned
    - Not concerned at all

53) How concerned are you that a vaccine might not prevent the disease?
    - Very concerned
    - Somewhat concerned
    - Neutral
    - Not too concerned
    - Not concerned at all

54) If you had another infant today, would you want him/her to get all the recommended vaccines?

- Yes
- No
- I don't know

55) Overall, how hesitant about childhood vaccines would you consider yourself to be?

- Very hesitant
- Somewhat hesitant
- Neutral
- Not too hesitant
- Not hesitant at all

56) I trust the information I receive about vaccines.

- Strongly disagree
- Disagree
- Neutral
- Agree
- Strongly agree

57) I am able to openly discuss my concerns about vaccines with my child's doctor.

- Strongly disagree
- Disagree
- Neutral
- Agree
- Strongly agree

58) All things considered, how much do you trust your child's doctor?

0 indicates being not sure at all and 10 being completely sure

- 0 Not sure at all
- 1
- 2
- 3
- 4
- 5
- 6

- ○ 7
- ○ 8
- ○ 9
- ○ 10 Completely sure

Thank you for your time!

موقف الوالدين تجاه تطعيمات الأطفال في دولة الإمارات العربية المتحدة.

نحن مجموعة من طلاب الطب في جامعة الشارقة نقوم بإجراء مشروع بحثي حول "موقف الوالدين تجاه تطعيمات الأطفال في دولة الإمارات العربية المتحدة"، والغرض من هذه الدراسة هو تقييم رأي الوالدين تجاه التطعيمات التي يتلقاها أطفالهم في دولة الإمارات العربية المتحدة.

لقد تم اختياركم بشكل عشوائي للمشاركة في هذه الدراسة ومشاركتكم طوعية تماماً. إن وافقتم على المشاركة، سيُطلب منكم ملء استبيان سيأخذ 5 إلى 7 دقائق من وقتكم.
لا توجد مخاطر متعلقة بالمشاركة في هذه الدراسة. الاستبيان مجهول الهوية، ونؤكد لكم أن ردودكم ستكون سرية وسيتم استخدامها فقط لأغراض البحث.
لكم الحرية للانسحاب قبل وأثناء الاجابة على الاستبيان؛ ولكن بمجرد إرسالكم إجاباتكم، لن نتمكن من إزالتها من بياناتنا بسبب طبيعة الدراسة السرية.

لأي استفسار عن الدراسة والنتائج المتعلقة بالدراسة، الرجاء التواصل مع مشرفة البحث الدكتورة هبة برقاوي على **hbarqawi@sharjah.ac.ae** أو 06-5057268.

في حال وجود أي مخاوف أو شكاوي بخصوص مشاركتك في هذا البحث ، يمكنك التواصل مع لجنة الأخلاقيات البحثية على الرقم 06505730 او عبر البريد الإلكتروني
REC@sharjah.ac.ae

شكراً مقدماً على مشاركتكم.

يرجى الضغط على المربع أدناه لمتابعة الاستبيان
لقد قمت بقراءة المعلومات السابقة و أوافق على المشاركة في هذا البحث.

1) هل تعيش في دولة الإمارات العربية المتحدة؟
   a) نعم
   b) لا

2) هل لديك أي أطفال (حاليا أو سابقا)؟
   a) نعم
   b) لا

المعلومات الشخصية

3) الجنس: ---------------
4) العمر: ---------------
5) أعلى مستوى تعليمي تم الحصول عليه
   a) مدرسة إعدادية أو أقل
   b) مدرسة ثانوية
   c) شهادة بكالوريوس/ دبلوم مهني
   d) دراسات عليا (ماجستير/ دكتوراه/ إلخ..) أو أعلى

6) الحالة الاجتماعية
   a) متزوج/ متزوجة
   b) مطلق/ مطلقة
   c) أرمل/ أرملة

7) الجنسية
   a) مواطن إماراتي
   b) جنسية عربية أخرى
   c) جنسية غير عربية

8) مكان الإقامة
   a) أبوظبي
   b) دبي

c) الشارقة
d) عجمان
e) أم القيوين
f) رأس الخيمة
g) الفجيرة

9) مجال العمل
a) عامل في القطاع الصحي (طبيب، ممرض، طبيب أسنان، صيدلي، إدارة الرعاية الصحية، إلخ..)
b) عامل في مجال آخر غير طبي
c) طالب (العلوم الصحية، طب، طب أسنان، إلخ..)
d) طالب (تخصص غير طبي)
e) ربة منزل
f) عاطل عن العمل (خلفية طبية)
g) عاطل عن العمل (خلفية غير طبية)

10) هل أخذت تطعيم كوفيد-19؟
a) لا
b) نعم، جرعة واحدة
c) نعم، جرعتان
d) نعم، 3 جرعات
e) نعم، 4 جرعات أو أكثر

11) هل أخذت تطعيم الأنفلونزا العام الماضي؟
a) نعم
b) لا

12) :عدد الأطفال في الأسرة
a) 1
b) 2
c) 3
d) 4 أو أكثر

13) أعمار الأطفال في الأسرة

حدد كل ما ينطبق

a) 1 - 11 شهر
b) سنة - سنتين
c) 3 سنوات - 5 سنوات
d) 6 سنوات - 11 سنة
e) 12 سنة - 18 سنة
f) 19 سنة أو أكبر

14) هل لدى أطفالك تأمين صحي؟

a) نعم، كلهم لديهم تأمين
b) نعم، ولكن فقط البعض منهم لديه تأمين
c) لا، لا أحد منهم لديه تأمين

15) هل حصل طفلك/أطفالك على التطعيمات الروتينية التي أقرتها وزارة الصحة في دولة الإمارات العربية المتحدة في مرحلة الطفولة؟

التطعيمات الإلزامية هي تلك التي تشكل جزءًا من برنامج التطعيمات الوطني لدولة الإمارات العربية المتحدة

a) نعم، كلهم حصلوا على التطعيمات
b) نعم، ولكن البعض منهم فقط حصل على التطعيمات
c) لا، لم يحصل أي منهم على التطعيمات
d) غير متأكد

16) هل تلقى طفلك/أطفالك تطعيمات غير تلك التي حددتها وزارة الصحة في دولة الإمارات العربية المتحدة؟

هذه التطعيمات ليست جزءًا من برنامج التحصين الوطني لدولة الإمارات العربية المتحدة ولكن قد يوصي بها الطبيب.

a) نعم، كلهم حصلوا على التطعيمات
b) نعم، ولكن البعض منهم فقط حصل على التطعيمات
c) لا، لم يحصل أي منهم على التطعيمات
d) غير متأكد

17) أي مما يلي هي المخاوف التي كانت لديك/لديك فيما يتعلق بتطعيمات الأطفال؟

حدد كل ما ينطبق.

a) مؤلمة وتسبب الحمى.

b) يعطى الكثير من التطعيمات في زيارة واحدة للطبيب.
c) قد تسبب التطعيمات صعوبات في التعلم (مثل التوحد).
d) المكونات الموجودة في التطعيمات غير آمنة.
e) تُعطى التطعيمات للأطفال للوقاية من الأمراض غير الخطيرة.
f) تُعطى التطعيمات للأطفال للوقاية من الأمراض التي من غير المحتمل أن يصابوا بها.
g) قد تسبب التطعيمات أمراضًا مزمنة (مثل مرض السكري والربو).
h) لدي مخاوف أخرى بشأن تطعيمات الأطفال.
i) ليس لدي أي مخاوف بشأن تطعيمات الأطفال.

18) كم مرة تقوم بزيارة طبيب طفلك/أطفالك لإجراء الفحوصات الدورية؟
a) شهرياً
b) كل 3 أشهر
c) كل 6 أشهر
d) سنوياً
e) غير ذلك

19) لا أقوم بزيارة طبيب أطفالي لإجراء الفحوصات الدورية

موقفك تجاه تطعيم الأطفال ومصادر المعلومات

20) ما مدى أهمية تطعيم الأطفال ضد كل من الأمراض التالية؟

- مرض الحصبة:
a) مهم جداً
b) مهم نوعاً ما
c) ليس مهماً جداً
d) ليس مهماً على الإطلاق
e) لا أعرف

- التهاب السحايا:
f) مهم جداً
g) مهم نوعاً ما
h) ليس مهماً جداً

i) ليس مهماً على الإطلاق
j) لا أعرف

- السعال الديكي:
k) مهم جداً
l) مهم نوعاً ما
m) ليس مهماً جداً
n) ليس مهماً على الإطلاق
o) لا أعرف

- مرض فيروس الروتا:
p) مهم جداً
q) مهم نوعاً ما
r) ليس مهماً جداً
s) ليس مهماً على الإطلاق
t) لا أعرف

- كوفيد-19:
u) مهم جداً
v) مهم نوعاً ما
w) ليس مهماً جداً
x) ليس مهماً على الإطلاق
y) لا أعرف

- الانفلونزا:
z) مهم جداً
aa) مهم نوعاً ما
bb) ليس مهماً جداً
cc) ليس مهماً على الإطلاق
dd) لا أعرف

اذكر إلى أي مدى توافق أو لا توافق على العبارات التالية:

21)تطعيمات الطفولة مهمة لصحة طفلي.

a) موافق بشدة
b) موافق
c) محايد
d) غير موافق
e) غير موافق بشدة

22)تطعيمات الأطفال فعَّالة.

a) موافق بشدة
b) موافق
c) محايد
d) غير موافق
e) غير موافق بشدة

23)إن تطعيم طفلي أمر مهم لصحة الآخرين في مجتمعي.

a) موافق بشدة
b) موافق
c) محايد
d) غير موافق
e) غير موافق بشدة

24)جميع تطعيمات الأطفال التي يقدمها البرنامج الحكومي في مجتمعي مفيدة.

a) موافق بشدة
b) موافق
c) محايد
d) غير موافق
e) غير موافق بشدة

25)تحمل التطعيمات الجديدة مخاطر أكبر من التطعيمات القديمة.

a) موافق بشدة

b) موافق
c) محايد
d) غير موافق
e) غير موافق بشدة

26) المعلومات التي أتلقاها حول التطعيمات من برنامج التطعيمات موثوقة وجديرة بالثقة.

a) موافق بشدة
b) موافق
c) محايد
d) غير موافق
e) غير موافق بشدة

27) إن الحصول على التطعيمات هو وسيلة جيدة لحماية طفلي/أطفالي من المرض.

a) موافق بشدة
b) موافق
c) محايد
d) غير موافق
e) غير موافق بشدة

28) بشكل عام، أفعل ما يوصي به طبيبي أو مقدم الرعاية الصحية فيما يتعلق بالتطعيمات لطفلي/أطفالي.

a) موافق بشدة
b) موافق
c) محايد
d) غير موافق
e) غير موافق بشدة

29) أنا قلق بشأن الآثار السلبية الخطيرة للتطعيمات.

a) موافق بشدة
b) موافق
c) محايد
d) غير موافق

e) غير موافق بشدة

30) لا يحتاج طفلي/أطفالي إلى تطعيمات للأمراض التي لم تعد شائعة بعد الآن.

a) موافق بشدة
b) موافق
c) محايد
d) غير موافق
e) غير موافق بشدة

الأسئلة التالية تتعلق بمعرفتك بما يتعلق بتطعيمات الأطفال

31) أجد أن مستوى معرفتي بتطعيمات الأطفال هو:

a) ضعيف
b) غير كافي
c) كافي
d) جيد
e) ممتاز

32) هل تعتقد أن لديك مصادر كافية للمعلومات حول التطعيمات؟

a) نعم
b) لا

33) ما هي المصادر التي تستخدمها للحصول على معلومات حول تطعيمات الأطفال؟

a) عن طريق الآخرين
b) الطبيب العام/ طبيب الأطفال
c) الطبيب الاخصائي
d) وحدة صحة الأسرة
e) المواقع الحكومية (مركز مكافحة الأمراض والوقاية منها، وزارة الصحة، هيئة صحة دبي، دائرة الصحة، منظمة الصحة العالمية، إلى آخره)
f) المدونات الإلكترونية/المنتديات الإلكترونية/المواقع غير الحكومية
g) وسائل الإعلام (راديو/ تلفزيون/ صحف)

h) وسائل التواصل الاجتماعي
i) المدرسة/الجامعة
j) الحركات/المجموعات المعادية للتطعيمات
k) مصادر آخرى
l) لا أستخدم أي مصادر للحصول على معلومات حول تطعيمات الأطفال.

اذكر إلى أي مدى توافق أو لا توافق على العبارات التالية:

34) أجد أن المعلومات المتعلقة بالتطعيمات الموجودة على وسائل التواصل الاجتماعي والمنتديات الإلكترونية مفهومة.
a) أعترض
b) أعترض نوعاً ما
c) اوافق نوعاً ما
d) اوافق

35) أجد أن المعلومات المتعلقة بالتطعيمات الموجودة على المواقع الحكومية مفهومة.
a) أعترض
b) أعترض نوعاً ما
c) اوافق نوعاً ما
d) اوافق

36) يمكنني التعرف على الأخبار المزيفة المتعلقة بالتطعيمات
a) أعترض
b) أعترض نوعاً ما
c) اوافق نوعاً ما
d) اوافق

37) أنا أثق في المعلومات المتعلقة بالتطعيمات التي تقدمها المواقع الحكومية.
a) أعترض
b) أعترض نوعاً ما
c) اوافق نوعاً ما
d) اوافق

38) أنا أثق في المعلومات المتعلقة بالتطعيمات التي يقدمها الأطباء.

a) أعترض
b) أعترض نوعاً ما
c) اوافق نوعاً ما
d) اوافق

39) أجد أن المعلومات المتعلقة بالتطعيمات الموجودة على شبكات التواصل الاجتماعي صحيحة.

a) أعترض
b) أعترض نوعاً ما
c) اوافق نوعاً ما
d) اوافق

40) عندما أقرأ معلومات التطعيم عبر الإنترنت، أقوم بمراجعتها مع مصادر أخرى للتحقق من صحتها.

a) أعترض
b) أعترض نوعاً ما
c) اوافق نوعاً ما
d) اوافق

41) أعتقد أن المعلومات التي أجدها عبر الإنترنت قد تؤثر على قراري بالحصول على التطعيم.

a) أعترض
b) أعترض نوعاً ما
c) اوافق نوعاً ما
d) اوافق

الممارسات والمعتقدات المتعلقة بتطعيمات الأطفال

فيما يلي بعض الأسئلة الإضافية المتعلقة بمعتقداتك وممارساتك تجاه تطعيمات الأطفال.

لكل سؤال، يرجى اختيار الخيار الذي يصف وجهات نظرك أو معتقداتك أو ممارساتك على أفضل وجه

42) هل سبق وأن تأخرت عن إعطاء التطعيم لطفلك لأي سبب عدا المرض أو الحساسية؟

a) نعم

b) لا
c) لا أعرف

43)ما هو سبب التأخير؟
a) لم أتاخر عن إعطاء أي تطعيم لاسباب غير المرض أو الحساسية
b) عدم التوصية من قبل الطبيب
c) الخوف من إعطاء التطعيم لطفلك
d) النسيان
e) القلق من الآثار الجانبية
f) التطعيم لم يكن متوفرا في مركز التطعيم
g) أسباب أخرى

44) هل سبق وأن امتنعت عن إعطاء التطعيم لطفلك لأي سبب عدا المرض أو الحساسية
a) نعم
b) لا
c) لا أعرف

45)ما السبب الذي منعك من إعطاء التطعيم لطفلك؟
a) لم أمتنع عن إعطاء اي تطعيم لاسباب غير المرض أو الحساسية
b) عدم التوصية من قبل الطبيب
c) الخوف من إعطاء التطعيم لطفلك
d) النسيان
e) القلق من الآثار الجانبية
f) التطعيم لم يكن متوفرا في مركز التطعيم
g) أسباب أخرى

46)كم أنت متأكد من أن إتباع جدول التطعيمات الموصى به فكرة جيدة لطفلك؟

الرجاء اختيار الإجابة على مقياس من 0 إلى 10، حيث 0 هي "غير متأكد على الإطلاق" و**" 10 "** متأكد بشدة

a) 0 لا أثق على الإطلاق
b) 1
c) 2
d) 3
e) 4

f) *5*
g) *6*
h) *7*
i) *8*
j) *9*
k) 10 أثق بشكل كامل

47) يعطى الأطفال عدد تطعيمات زائد عن ما هو جيد لهم.

a) موافق بشدة
b) موافق
c) غير متأكد
d) غير موافق
e) غير موافق بشدة

48) أعتقد أن الكثير من الأمراض التي تقي منها التطعيمات هي أمراض خطيرة.

a) موافق بشدة
b) موافق
c) غير متأكد
d) غير موافق
e) غير موافق بشدة

49) من الأفضل لطفلي أن يحصل على مناعته عند إصابته بالمرض من أن يأخذ التطعيم.

a) موافق بشدة
b) موافق
c) غير متأكد
d) غير موافق
e) غير موافق بشدة

50) من الأفضل أن يحصل الأطفال على تطعيمات أقل كل مرّة.

a) موافق بشدة
b) موافق
c) غير متأكد

d) غير موافق
e) غير موافق بشدة

51) ما مدى مخاوفك من أن طفلك قد يصاب بآثار جانبية خطيرة من التطعيم؟
a) غير خائف تماماً
b) غير خائف
c) غير متأكد
d) خائف قليلاً
e) خائف كثيراً

52)ما مدى مخاوفك من أن إحدى تطعيمات الطفولة قد تكون غير آمنة؟
a) غير خائف تماماً
b) غير خائف
c) غير متأكد
d) خائف قليلاً
e) خائف كثيراً

53). ما مدى مخاوفك من أن التطعيم قد لا يمنع الإصابة بالمرض؟
a) غير خائف تماماً
b) غير خائف
c) غير متأكد
d) خائف قليلاً
e) خائف كثيراً

54) إذا لديك طفل آخر، هل تحرص على إعطائه كافة التطعيمات الموصى بها؟
a) نعم
b) لا
c) لا أعرف

55)عموماً، ما مدى ترددك حول إعطاء تطعيمات الطفولة؟
a) غير متردد تماماً

b) غير متردد
c) غير متأكد
d) متردد قليلاً
e) متردد كثيراً

56)أنا أثق بالمعلومات المقدمة لي عن التطعيمات.
a) موافق بشدة
b) موافق
c) غير متأكد
d) غير موافق
e) غير موافق بشدة

57)أنا قادر على مناقشة مخاوفي عن التطعيمات بكل صراحة.
a) موافق بشدة
b) موافق
c) غير متأكد
d) غير موافق
e) غير موافق بشدة

58)مع الأخذ بعين الاعتبار لكل الامور، ما مدى ثقتك بطبيب الأطفال في العيادة؟
الرجاء اختيار الإجابة حسب المقياس من 0 إلى 10، حيث 0 هي "عدم الثقة على الإطلاق" و 10تعني "الثقة بشدة
a) 0 لا أثق على الإطلاق
b) 1
c) 2
d) 3
e) 4
f) 5
g) 6
h) 7
i) 8
j) 9
k) 10 أثق بشدة

شكرًا لك على وقتك.
